# Shark and ray cartilage canals show blood vessels can exist in mature cartilage without endochondral bone formation

**DOI:** 10.1016/j.isci.2026.117871

**Published:** 2026-10-31

**Authors:** Benjamin Flaum, Ronald Seidel, Maximus Yeatman-Biggs, Theda I. Hinrichs, Jana Ciecierska-Holmes, Salman O. Matan, Emilio J. Gualda, Kady Lyons, Victoria Camilieri-Asch, Susan R. McGlashan, Laura Ekstrom, Lawrence Bonassar, Mélanie Debiais-Thibaud, Daniel Baum, Michael J. Blumer, Mason N. Dean

**Affiliations:** 1 Department of Infectious Diseases & Public Health, City University of Hong Kong, Hong Kong, China; 2 Department Biomaterials, Max Planck Institute of Colloids & Interfaces, Potsdam, Germany; 3 Department of Anatomy and Medical Imaging, Waipapa Taumata Rau University of Auckland, Auckland, New Zealand; 4 Max Planck Queensland Centre for the Materials Science of Extracellular Matrices, Queensland University of Technology, Brisbane, QLD, Australia; 5 School of Mechanical, Medical, and Process Engineering, Faculty of Engineering, Queensland University of Technology, Brisbane, QLD, Australia; 6 Humboldt-Universität zu Berlin, Institute of Biology, 10099 Berlin, Germany; 7 Meinig School of Biomedical Engineering, Cornell University, New York, NY, USA; 8 Department of Agri-Food Engineering and Biotechnology, Universitat Politècnica de Catalunya, 08860 Castelldefels, Spain; 9 Biological Sciences Department, California State University, Long Beach, CA, USA; 10 Department of Biological, Chemical, and Environmental Sciences, Wheaton College, Norton, MA, USA; 11 Institut des Sciences de l'Evolution, University Montpellier, CNRS, IRD, EPHE, Montpellier, France; 12 Department of Visual and Data-Centric Computing, Zuse Institute Berlin, Berlin, Germany; 13 Division of Clinical and Functional Anatomy, Medical University of Innsbruck, Mullerstrasse 59, 6010 Innsbruck, Tirol, Austria; 14 Centre for Nature Inspired Engineering (CNIE), City University of Hong Kong, Hong Kong, China

**Keywords:** comparative cartilage biology, skeletal tissue evolution, development, elasmobranch fishes, chondrocyte, tessellated cartilage

## Abstract

Cartilage in adult tetrapod skeletons is known to lack blood vessels. In young birds/mammals, however, a dense network of vasculature-containing tunnels—cartilage canals—perforates the growing skeleton, nourishing cartilage, and developing ossification centers for later bone. As the canals and their vascular network regress as the animals age, healthy cartilage in adults is typically regarded as avascular. We use diverse tissue characterization and visualization (e.g., light/electron microscopy, microCT) to show skeletons of adult rays/sharks (elasmobranchs) possess persistent cartilage canals. Elasmobranch canal morphology and tissue composition support a structural analogy with mammalian cartilage canals, suggesting that early jawed vertebrates had vasculature-invaded cartilage. However, the anatomical location of canals—extending away from, not toward, mineralized tissue—and the lack of endochondral ossification in ray/shark cartilage suggest functions divergent from amniotes. We describe distinctive features and variation in elasmobranch cartilage canals, discuss their potential for tissue mineralization, and implications for their presence in an animal clade with continuously growing cartilaginous skeletons.

## Highlights

• Vascular canals have been found in the adult cartilage of 25 elasmobranch species
• Elasmobranch cartilage canals are structurally analogous to tetrapod cartilage canals
• The elasmobranch cartilage canal network is simpler than tetrapod ones
• Cartilage canals are a basal adaptation in vertebrates or evolved multiple times

## Graphical abstract

**Figure:**
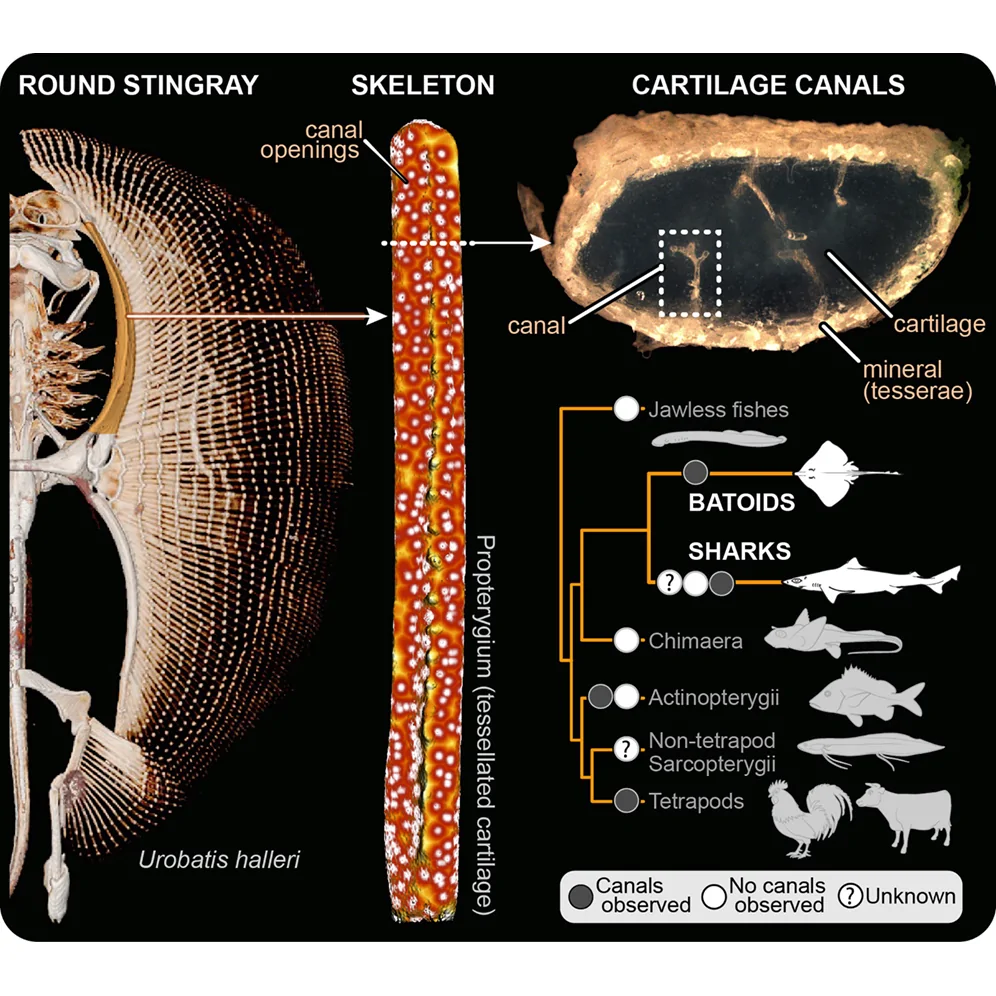

## Teaser

Anatomy; Physiology; Evolutionary biology; Developmental biology

## Introduction

Most bones in the skeleton of tetrapod animals form indirectly from cartilage precursors converted into bone during early development and postnatal growth.^1^^,^^2^ During this process, known as endochondral ossification, the cartilage cells (chondrocytes) enlarge, triggering mineralization of the surrounding cartilage matrix as well as the invasion of blood vessels and bone-related cells into the cartilage scaffold. The cartilage matrix is then degraded by matrix metalloproteinases (MMPs), resorbed by chondroclasts, and replaced by bone matrix (formed by osteoblasts), while the hypertrophic chondrocytes undergo cell death (apoptosis).^3^^,^^4^^,^^5^^,^^6^^,^^7^^,^^8^ In the development of long bones (e.g., femur, tibia), a primary ossification center is first formed in the mid-shaft (diaphysis), followed by the formation of secondary ossification centers at the ends of the bone (epiphyses). Vasculature is key in this tissue transformation. Yet, whereas in developing primary ossification centers blood vessels invade the cartilage directly, in long bone secondary ossification centers (but also foot bones and vertebrae), blood vessels are ferried into the reorganizing unmineralized matrix via tunnels called cartilage canals.^9^^,^^10^^,^^11^ Cartilage canals are invaginations of the fibrous layer (perichondrium) that covers cartilage, migrating into the epiphysis via the action of matrix metalloproteinases and vascular endothelial growth factor (VEGF, which attracts blood vessels).^12^^,^^13^^,^^14^ In this way, cartilage canals allow a pocket of the skeleton’s external environment to penetrate the cartilage and contribute crucially to the formation of the localized ossification centers that establish the adult skeleton’s bone. However, the canals in tetrapods recede during development, replaced by the expanding bone tissue. As a result, cartilage canals and the vasculature they carry are considered absent in the cartilage of adult vertebrate animals ^e.g^.^15^^,^^16^^,^^17^^,^^18^

Cartilage canals have been described in a range of terrestrial vertebrate species, especially mammals and reptiles (including birds) (see citations in Table S4). We show here, however, that cartilage canals—structurally analogous and potentially evolutionarily homologous to those of mammals—are also a primary component of the skeletons of adult rays and sharks and relatives (elasmobranch fishes), animals with skeletons made entirely of cartilage. As the cartilaginous skeletons of these fishes are never converted to bone, this suggests that cartilage canals may perform divergent functions across vertebrate lineages.

## Results and discussion

### In adult stingrays, as in young mammals, cartilage canals perforate the skeleton

The cartilage of rays, sharks and chimaeras is structurally unique among vertebrates in having a surface veneer of mineralized blocks called tesserae (Figures 1C–1F).^19^^,^^20^^,^^21^ In rays and sharks, tesserae are typically hundreds of microns wide and deep, sandwiched between the outer layer of fibrous perichondrium and the unmineralized cartilage in the core of the skeleton (the latter grossly similar to mammalian hyaline cartilage).^22^^,^^23^^,^^24^^,^^25^ The unmineralized cartilage, however, typically dominates ray and shark skeletons, with tesserae occupying a comparatively small proportion of skeletal cross-sectional area in most species (e.g., ∼10–20% in the Round stingray *Urobatis halleri*^26^^,^^27^). As the majority of the skeleton is tessellated cartilage (the composite of unmineralized cartilage and mineralized tesserae; Figure 1), we focus on this rather than the other largely mineralized tissue types restricted to the vertebral column.^19^^,^^28^^,^^29^^,^^30^

**Figure 1:**
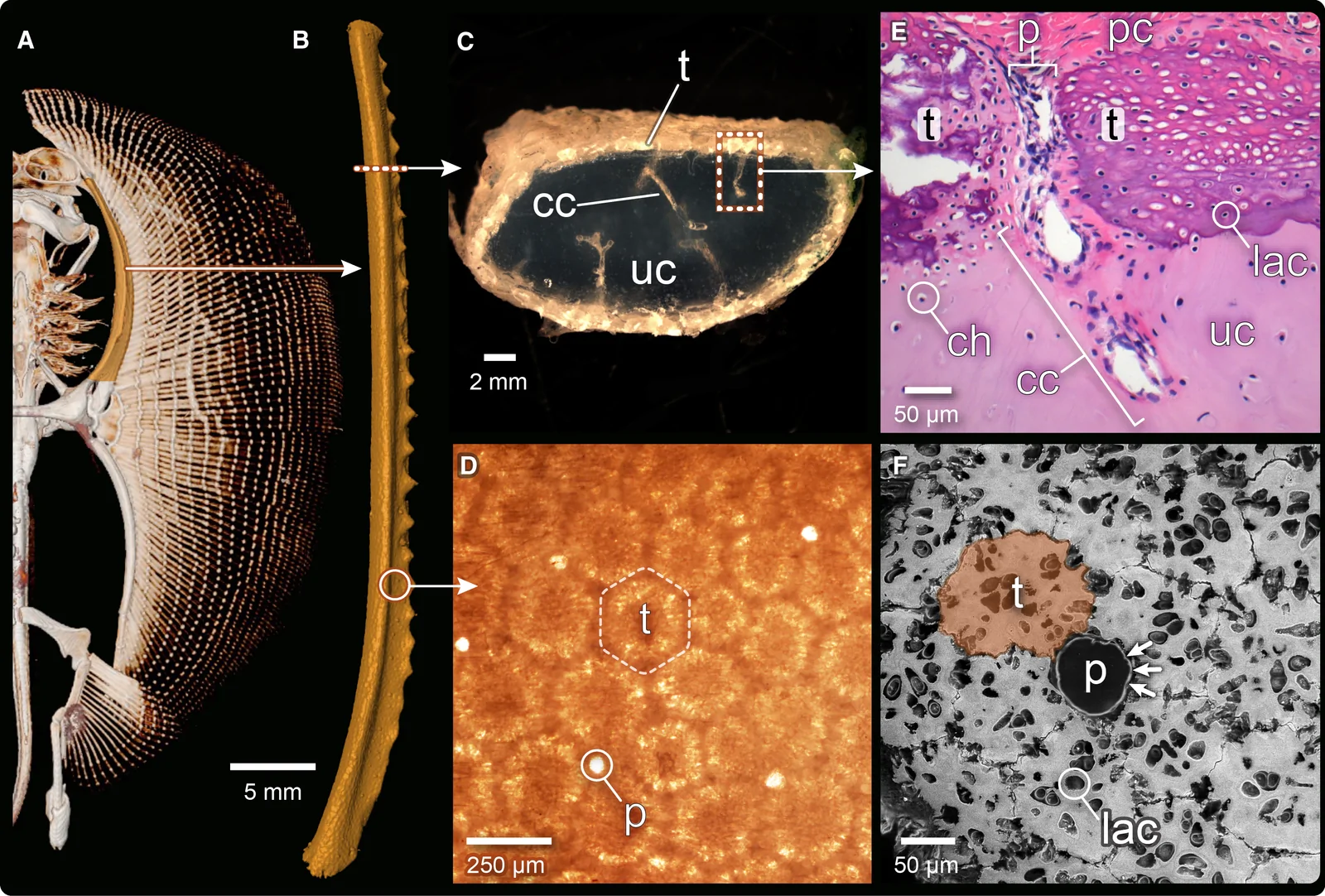
The tessellated cartilage of the Round stingray is perforated by cartilage canals The Round stingray (*Urobatis halleri*, A), like all rays and sharks, has a skeleton made predominantly of tessellated cartilage: unmineralized cartilage (uc) with surface mineralization. Individual skeletal elements (e.g., the propterygium, B), when cross-sectioned (C), show an outer veneer of mineralized blocks (tesserae, t), which are polygonal when viewed from the skeletal surface (D and F). At the junctions of some tesserae, large pores (p) lead into cartilage canals (cc), which pass from the perichondrium (pc), between tesserae, and into the underlying cartilage (D–F). The fibrous lining of canals/pores is visible in E (darker pink staining) and F (arrows), as are the chondrocytes (ch) within the unmineralized cartilage (E) and the cell lacunae (lac) within tesserae (F). Note that the darker pink staining around the canal in E is continuous with the fibrous perichondrium (see also Figure 3C). A,B: μCT; C,D: light microscopy; E: H&E histology; F: BSE-SEM. All images are from *U. halleri*, except C from *Leucoraja erinacea*. (Scale bars, B = 5 mm; C = 2 mm; D = 250 μm; E, F = 50 μm)

We first observed cartilage canals in dissections of adult Round stingray (Figure 1),^31^ a small species we have used as a model for characterization of tesserae microanatomy and materials ^e.g^.^22^^,^^23^^,^^24^^,^^25^^,^^32^ In this species, cartilage canals were visible with the naked eye in cross-sectioned skeletal specimens as white streaks or tubular gaps in the otherwise glassy cartilage matrix, however only once they had filled with a material of a different refractive index than the surrounding matrix (e.g., blood or air; see Figure S1). This optical behavior may explain why canals have almost entirely escaped notice in more than one hundred years of anatomical literature (but see^29^^,^^31^^,^^33^^,^^34^^,^^35^). We characterized the cartilage canals of the Round stingray using a variety of tissue preparations and imaging approaches (e.g., unstained samples in light microscopy, stained histological samples, cryo-scanning electron microscopy); in diverse pieces of the skeleton; and in both sub-adult and adult specimens of varying ages (Table S3).

In the Round stingray, as in mammals, cartilage canals originate from the perichondrium and end blindly in the unmineralized cartilage core of the skeleton (Figure 1C–1E); this basic topology was confirmed in many other elasmobranch species we examined (see later in discussion and Figures 4 and S4; Table S3). Passing from the perichondrium to the unmineralized cartilage, stingray canals did not bore through individual tesserae but rather traversed the mineralized layer via large pores (∼30–60 μm wide; Figures 1D–1F and S2; Table S1), found at the meeting points of multiple tesserae (Figures 1D–1F). Canal pores were irregularly spaced (515.7 ± 144.3 μm apart in a 6.2 × 2.0 mm ROI; Table S1; Figures 2A and S2) and strikingly obvious in light microscopy, electron microscopy (EM), and microCT preparations, being considerably larger and more circular than other joints or gaps between tesserae (Figure 1D–1F). Canal pores are therefore likely the intertesseral pores described by previous authors,^23^^,^^27^^,^^36^^,^^37^^,^^38^^,^^39^ although their role as conduits for cartilage canals was never realized.

### Stingray canals are prevalent, but simply structured and not linked to mineralization

Taking advantage of the fibrous lining of canals (see later in discussion), we treated a long piece of the Round stingray wing skeleton with phosphotungstic acid (PTA), enhancing tissue contrast to make canals visible against the unstained cartilage in microCT scans (Figure 2). We applied a custom semi-automatic segmentation approach, thresholding, and digitally isolating canals, allowing the entire population to be visualized and quantified (408 canals within a skeletal element ∼5 cm long and ∼4 mm wide/thick) (Figures 2A and 2B). To our knowledge, no comparable quantitative 3D map of tetrapod canals exists; however, images and 3D renderings from mammal and chicken cartilage show that canals are typically tens of microns in diameter and form a dense and richly dendritic network in the epiphysis, before the development of secondary ossification centers^40^^,^^41^^,^^42^^,^^43^ (Figures 2D and 2E). In tetrapod long bone epiphyseal cartilage, canals can be several millimeters long, extending sometimes nearly the full cartilage thickness to the deep hypertrophic zone of the epiphyseal growth plate.^40^^,^^41^ In contrast, stingray cartilage canals are far simpler in extent and form (Table S2), occupying only ∼0.3% of the cartilage volume, having a near-constant diameter (24.1 ± 5.3 μm), similar to tetrapod canals, but being mostly unbranched (1.06 ± 0.34 nodes), with bi- and trifurcated canals also rarely present (only ∼6% of canals) (Figures 2B and 2C; Table S2). Stingray canals were comparatively straight (tortuosity = 1.08 ± 0.08), running either parallel to the underside of tesserae or extending perpendicular to them, deeper into the cartilage (Figure 2B). In contrast to tetrapod epiphyses (e.g., Figures 2D and 2E), no stingray canals traversed the entire cartilage bulk, instead ending <50% through the skeleton’s thickness (∼453.3 ± 337.9 μm long; Figure 2B; Table S2). Stingray canals are more densely distributed on average than those of newborn canine pup skeletons (571.42 ± 176.79 μm vs. ∼1.4 mm apart, respectively^44^^,^^45^; Table S2), with apparent variability between lateral and medial surfaces of the propterygium, suggesting a degree of regionalization (Figure 2A).

**Figure 2:**
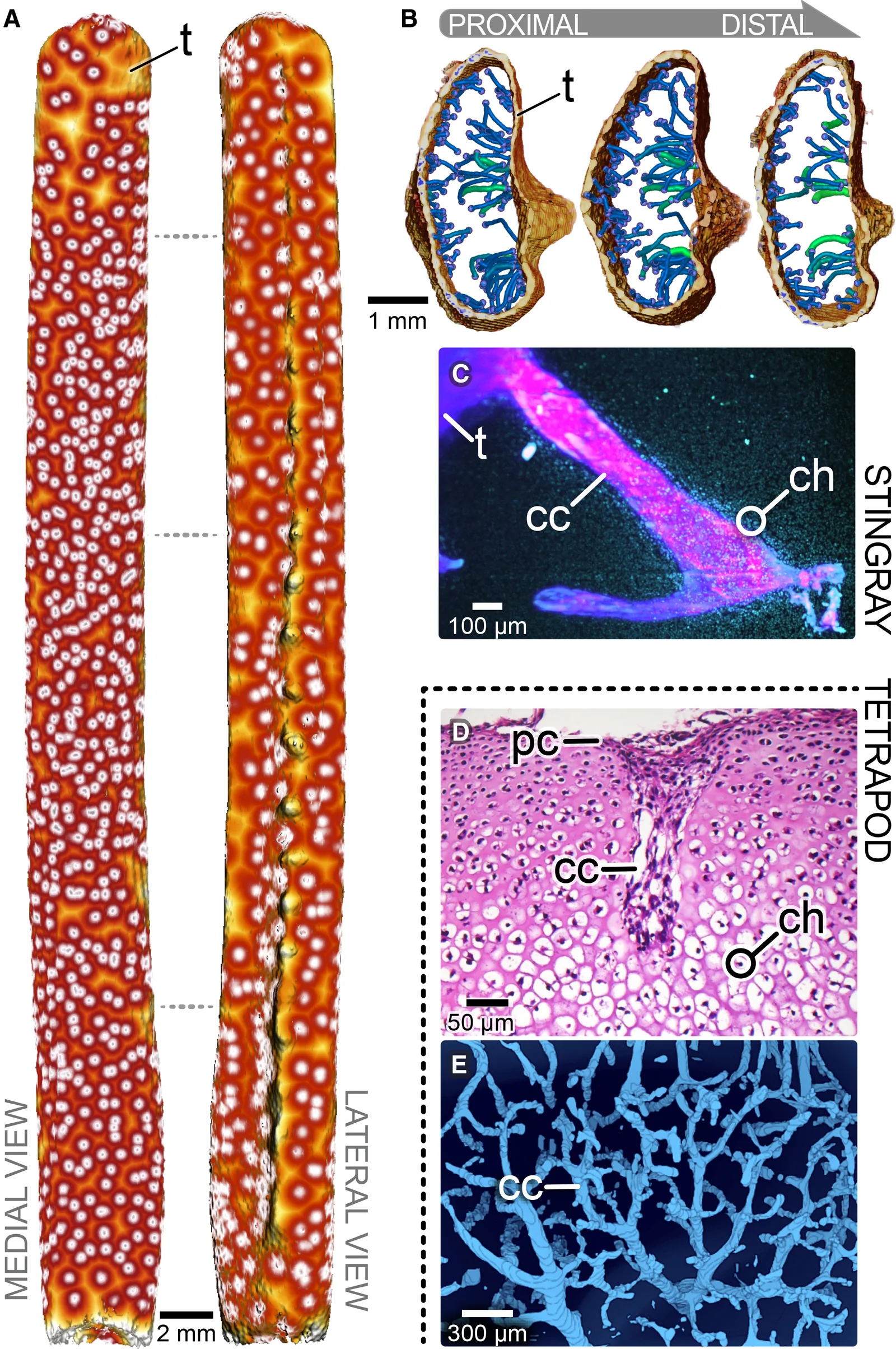
The cartilage canal network in the skeleton of Round stingray is pervasive, but less complex than tetrapod networks On the outer surface of the propterygium of the Round stingray (A), the origins of canals (marked by white dots) are densely arrayed on both the medial (left) and lateral (right) mineralized surfaces (tesserae, t). Skeletonized renderings of canals in three cross sections of the same propterygium (B), taken from the locations of the dashed horizontal lines in A and with canals color-coded for diameter—using a gradient from blue (11 μm) to green (30 μm)—show canals that are largely similar in width but not length (see Table S2), with none passing entirely through the cartilage. Stingray cartilage canals (cc) are predominantly unbranched, but branching canals were also seen (C), and in all cases chondrocytes (ch) were found clustered tightly around canals in the matrix. In contrast, tetrapod canals (D and E) are only found in growing cartilage (D: note the high chondrocyte density in young D7 mouse epiphysis) and exhibit convoluted, dendritic networks (E: chicken e20 embryo epiphysis). A and B: quantitative μCT renderings; C: light sheet microscopy (tissue autofluorescence); D: H&E histology; E: 3D reconstruction from histology (modified with permission from^40^). (Scale bars, A = 2 mm; B = 1 mm; C = 100 μm; D = 50 μm; 300 μm = E)

Despite the gross similarity between elasmobranch and tetrapod canals as ingress points into the cartilage bulk, their trajectories are notably different. In elasmobranch fishes, all margins of tesserae are thought to be active mineralization fronts, allowing tesserae to thicken and widen during development by accretion of new mineralized tissue layers.^19^^,^^22^^,^^23^^,^^24^ By perforating the tesserae layer to reach the uncalcified cartilage, elasmobranch canals therefore extend away from mineralizing zones rather than toward them, contrasting the association of tetrapod canals with ossification centers.^13^ This fundamental difference questions whether the canals of elasmobranchs and tetrapods are functionally and compositionally comparable.

### Stingray canals are structurally analogous to those of tetrapods

As invaginations of the perichondrium, tetrapod cartilage canals are tubular conduits lined with perichondrial Type-1 collagen, penetrating deep into the mineralizing cartilage matrix, carrying mesenchymal cells, unmyelinated nerve fibers, macrophages, TRAP-positive cells (e.g., chondroclasts), and vasculature embedded in loose connective tissue.^14^^,^^41^^,^^45^^,^^46^^,^^47^ Similarly, our histology and EM preparations of stingray cartilage show a collar of fibrous material lining the canals (Figure 3) and immunohistochemistry (IHC), verified that this is Type-1 collagen, whereas the surrounding cartilage matrix is patterned on a cartilage-typical Type-2 collagen (Figures 3C–3F). The canal sheath is therefore an intrusion of Type-1 collagen into a Type-2-dominant tissue, similar to the mixing of collagens observed in other elasmobranch tissues.^25^^,^^48^^,^^49^ We saw no evidence of nerve fibers or TRAP-positive cells (e.g., breaking down the tesserae’s mineralized matrix). Importantly, however, stingray cartilage canals contain capillaries enmeshed in loose connective tissue (Figures 3B–3D, 3F, and S3), debunking the long-standing beliefs that the elasmobranch skeleton is avascular and/or inhibits vascular invasion.^1^^,^^20^^,^^50^^,^^51^ Adult stingray canals therefore, surprisingly, mirror canal composition in juvenile tetrapods, representing a perichondrial microcosm that provides entrance for vasculature into the skeleton. This demonstration challenges the textbook dogma that adult healthy cartilage lacks vasculature ^e.g^,^15^^,^^16^^,^^17^^,^^18^ suggesting an entirely different physiological environment for the adult cartilage of stingrays relative to that of other vertebrates.

**Figure 3:**
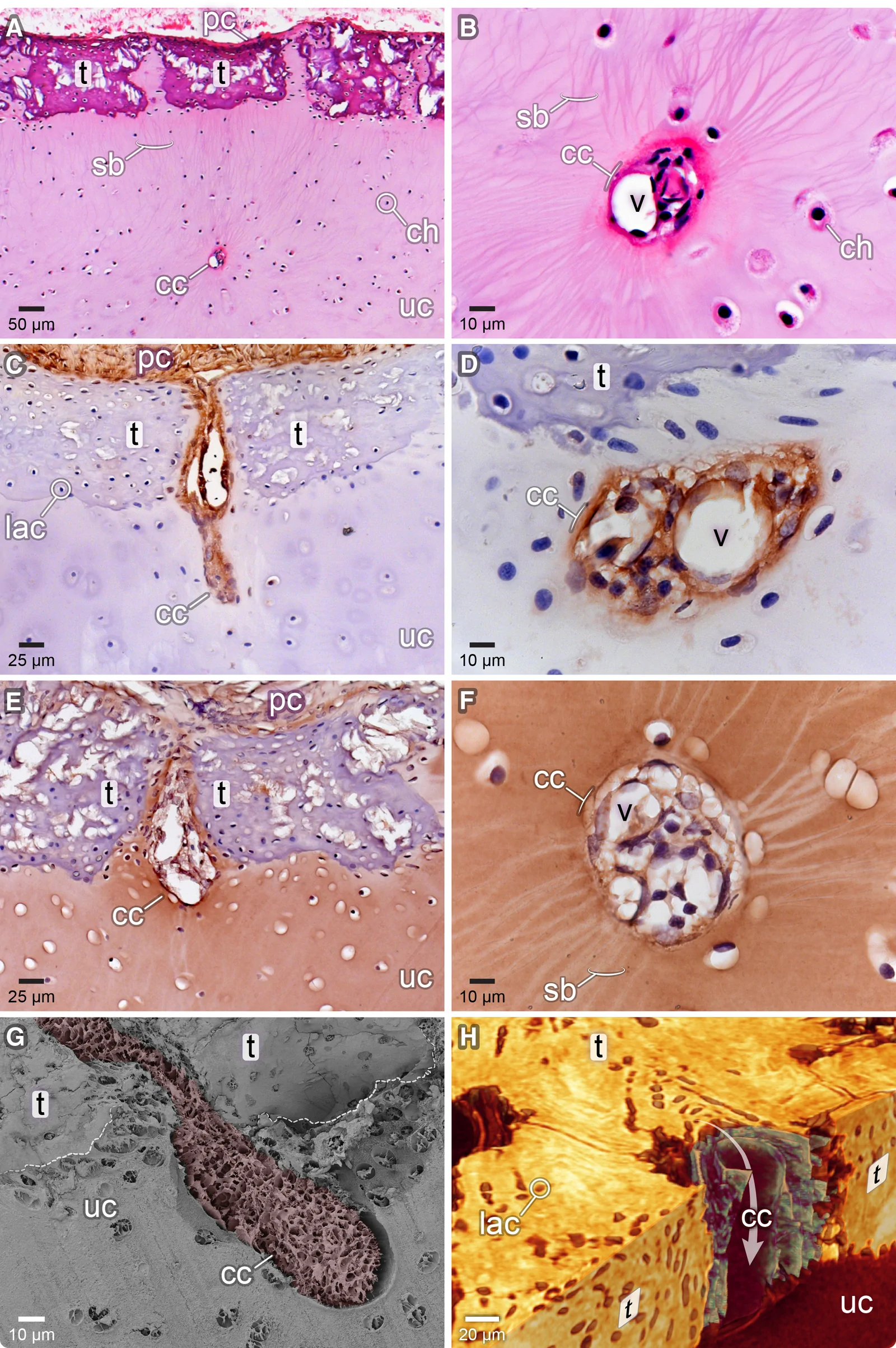
The histology of Round stingray cartilage canals shows them to be structurally analogous to mammalian ones In cross section within the unmineralized cartilage (uc) matrix, cartilage canals (cc) are roughly circular (A, B, D, and F) and contain vessels (v; see also Figure S3), with chondrocytes (ch) and threadlike *Saftbahnen* (sb) visible surrounding the canals. Immunohistochemistry (brown staining) shows that canals are invaginations of Type-1 collagen (stained brown in C, D) from the perichondrium (pc), whereas the unmineralized cartilage matrix is Type-2 collagen-based (E and F). The canal’s lining is therefore structurally distinct from the surrounding cartilage matrix, as shown in a freeze-fractured sample (G) of a longitudinally sectioned canal with the fibrous lining intact (i.e., the Type-1 collagen layer is viewed from its peripheral aspect, the canal lumen and its contents are not visible). A virtual section of μCT data (H)—with tesserae (t) sectioned in-plane on the upper left and a canal and two tesserae sectioned vertically on the bottom right—illustrates how the orientations of cell lacunae (lac) track the shape of canals in two planes (see also^36^). A and B: H&E histology; C,D: Type-1 collagen IHC; E,F: Type-2 collagen IHC; G: cryoSEM with canal lining pseudocolored; H: μCT with tesserae, canal lining, and other non-mineralized background (including lacunae within tesserae) pseudocolored yellow, green, and red, respectively. (Scale bars, A = 50 μm; B, D, F, G = 10 μm; C, E = 25 μm; H = 20 μm)

### Cartilage canals are widespread features in shark and ray skeletons

To verify that cartilage canals were not unique to the Round stingray among elasmobranch fishes, we assembled observations from a broad phylogenetic range of shark and ray species, many from specimens collected for other studies, which provided skeletal cross sections for evaluation (Table S3; Figures 4 and S4). As a result, age could not be controlled for and sampling location was not consistent, but tended to be primarily in the visceral arches (jaw, hyoid, branchial arches), as well as the pectoral/pelvic girdles and chondrocranium. Given that multiple canals were typically visible in each skeletal cross section from the Round stingray, we coded species as having canals if any (i.e., ≥1) could be definitively observed across several specimen sections. Larger canals that pass through the vertebral body of some large sharks were excluded,^52^ as they run only through a thick, highly mineralized tissue called ‘areolar cartilage’.^28^^,^^30^^,^^53^

Whereas cartilage canals were observed in all 17 examined species of batoid fishes (rays, skates, guitarfishes), canal presence was inconsistent in the 17 shark species investigated, with some families seen to contain a mix of species with and without canals (e.g., Carcharhiniformes, Lamniformes) (Figure 4). Additionally, canals were not observed in chimera, lamprey, or hagfish, species included as outgroup comparisons. Allocation of certain species as lacking canals is not a definitive demonstration of canal absence in those elasmobranch families; further systematic evaluation is required. However, the variation in the frequency of canal occurrence suggests that canals may be more physiologically important in some groups. Concomitantly, canals also showed a range of morphologies: whereas most batoid species had canal morphologies similar to the Round stingray’s, two sharks from different families (Common thresher *Alopias vulpinus* and Sandbar shark *Carcharhinus plumbeus*; Figures 4C and S1) had short and squat canals, while Angel sharks (*Squatina* spp.) had particularly elaborate canal systems (Figure 4B). This variation in cartilage canal network architecture suggests diversity in canal role and function (e.g. associated with different ecologies).

**Figure 4:**
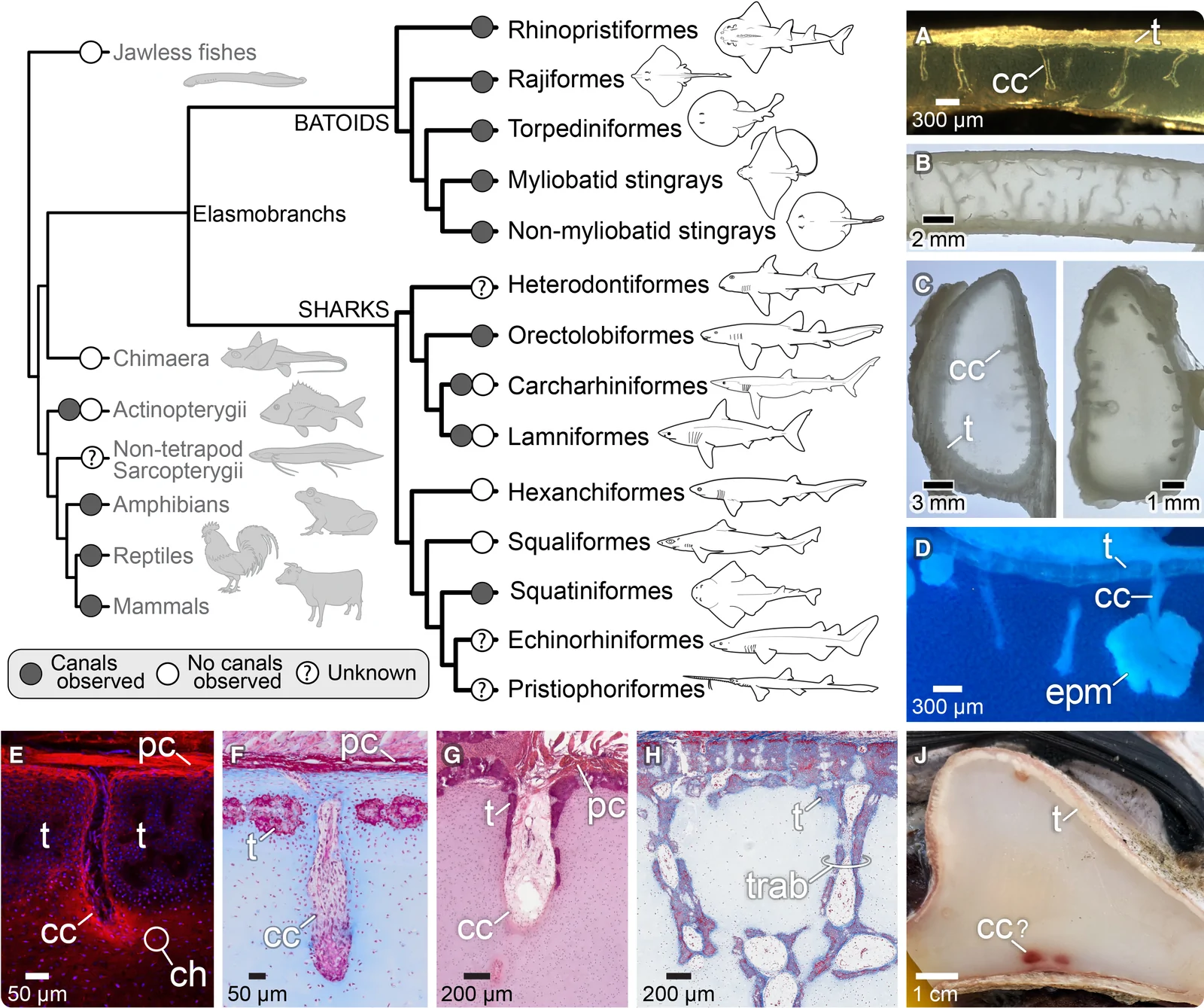
A phylogenetic survey of cartilage canal presence in elasmobranch fishes suggests that they are widespread in batoid fishes, but more patchily distributed in sharks Combined with our data for outgroups (chimaera, jawless fishes) and literature reports for other vertebrates (see Tables S3 and S4), current data suggest cartilage canals arose before or at least without a link to endochondral ossification. Circles at branch tips indicate whether canals were observed in the elasmobranch family members we examined or have been shown in the literature for any species of a group (see Tables S3 and S4); question marks indicate groups for which no data currently exists. Images of cartilage canals (cc) from various elasmobranch species (A–J, Table S3; Figure S4) illustrate that all instances show a similar arrangement, extending from the perichondrium (pc) between tesserae (t) to end in the unmineralized cartilage. Species, however, show a wide morphological variation, including sparsely branched canals similar to those in Round stingray (A: Batoid, *Leucoraja erinacea*); denser multibranched canals (B: Squalean shark, *Squatina squatina*); and short bulbous canals (C: Galean sharks, *Isurus oxyrinchus* (left) and *Carcharhinus plumbeus* (right)). Endophytic masses (epm) were on occasion seen associated with some canals (D: Batoid, *Urobatis halleri*) and chondrocytes (ch) were seen clustered around all canals examined at histological resolutions (e.g., E: Batoid, *Mobula tarapacana*; F: Galean shark, *Galeorhinus galeus*). In some species, canals show varying degrees of lining by tesserae, from partial coverage (G: Batoid, *Dasyatis pastinaca*) to fully covered trabeculae (trab) (H: Batoid, *Rhinoptera bonasus*). In the samples we had from some species, no canals were observed (see Table S3); in the case of the Basking shark (J: Galean shark, *Cetorhinus maximus*), canal presence was ambiguous, despite the massive thickness of the cartilage. See Figure S4 for additional images of canals from examined species not shown here. (Scale bars, A, D = 300 μm; B = 2 mm; C = 1 mm (L), 3 mm (R); E, F = 50 μm; G, H = 200 μm; and J = 1 cm).

### Implications

Tetrapod cartilage canals have three major putative functions. First, canals nourish growing epiphyseal cartilage, which would otherwise depend exclusively on passive diffusion of nutrients from capillary networks external to the skeleton.^54^ Perfusion examinations^44^ revealed that each canal contains a central arteriole that branches into a capillary glomerulus from which venules return to the perichondrium, thereby supplying the cartilage with nutrients and eliminating waste products. At the same time, an interruption of the cartilage canals’ blood supply by minor trauma can be an initiating factor of osteochondrosis, where ossification of the epiphysis is impaired, and necrotic islands of cartilage are retained.^55^^,^^56^^,^^57^ Second, several studies suggest that canals contain Type-2 collagen-synthesizing cells, which are responsible for the interstitial growth of the epiphysis.^41^^,^^46^^,^^54^^,^^58^^,^^59^ In piglets and foals, this occurs via chondrification, where canals regress at their distal ends, their endothelial cells apoptose, and their fibroblasts transdifferentiate into matrix-producing chondrocytes.^55^^,^^56^^,^^57^^,^^60^^,^^61^ Lastly, fibroblasts and mesenchymal cells in the connective tissue filling canals also express several bone-related proteins (Type-1 collagen, periostin, alkaline phosphatase, and Runx2), suggesting that they may later differentiate into bone cells (osteoblasts and osteocytes), which are crucial for the mineralization of the bone organic matrix (type-1 collagen) and thus formation of the secondary ossification center.^9^^,^^54^^,^^62^^,^^63^^,^^64^

The vast majority of cartilage canal research has focused on only a few species of mammals and birds (Table S4), where the canals have very similar structural characteristics. Within elasmobranchs, our demonstration of cartilage canals in a wide diversity of lineages suggests they are an ancestral feature for the clade with high potential for secondary loss (and maybe secondary gains). Mapping the presence of canals onto the vertebrate phylogeny (Figure 4), following the limited published species data (Tables S3 and S4), shows their documented occurrence in three disparate lineages: in elasmobranch fishes, tetrapods, and sturgeon. Two equally parsimonious evolutionary hypotheses are that canals either (1) arose once at the base of jawed vertebrates (gnathostomes) or (2) evolved independently at least three times (in elasmobranchs, in actinopterygian fishes and in tetrapods); in both scenarios, canals would have been patchily lost in some groups. These hypotheses should be tested, for instance, by exploring the degree to which developmental processes and molecular pathways are shared between elasmobranch and tetrapod cartilage canals. In addition, similar structural and developmental analyses should be performed in sturgeon to unite observations among three lineages of jawed vertebrates. If the “single origin” scenario is robustly supported, then one of the major anatomical features that permit conversion of cartilage into bone was already present in stem jawed vertebrate lineages, but likely only later co-opted into an ossification function in tetrapods.

Since chondrocytes rely on diffusion of nutrients through the gel-like extracellular matrix, it is thought that they must remain local to vasculature; in newborn canine pup skeletons, chondrocytes are never more than ∼700 μm away from canals.^44^^,^^45^ This constraint imposes profound limits on both the cell density and the size of cartilage elements in adult tetrapods, where healthy cartilage typically lacks blood vessels: in the femoral condyles of land tetrapods, articular cartilage thickness increases with negative allometry relative to body mass, with the largest species studied (e.g., elephant, rhinoceros, horse) having cartilage just ∼2–3 mm in thickness, with cell density decreasing greatly further away from the articular surface.^58^^,^^65^^,^^66^ There are no comparable datasets spanning the diversity of sharks and rays, but our dissections of various species regularly revealed skeletal cross sections centimeters in thickness, with some specimens of Basking shark (*Cetorhinus maximus*) cartilage exceeding 4–5 cm thick (oddly, however, canals could not be unequivocally demonstrated in this species, nor in two other large coldwater sharks, *Somniosus* and *Hexanchus*; Figure 4J; Table S3). Although a cartilaginous skeleton is a unifying characteristic of all sharks and rays, elasmobranch fishes are diverse, with >1200 species exhibiting a rich range of ecologies and habitats and a huge range of body sizes, from tens of centimeters to >10 m in length.^67^^,^^68^^,^^69^ The presence of canals may have released a maximum size constraint^69^ on elasmobranch cartilaginous skeletal elements: most canals we observed did not pass completely through the cartilage bulk, yet with the perichondrium surrounding the entire skeleton (unlike mammalian epiphysis cartilage), canals can enter—and supply tissue—from any direction.

Elasmobranch cartilage canals are tightly associated with chondrocytes. Where canals pass into the skeleton, cells within tesserae are oriented to follow the canal’s path (Figure 3H) and are in significantly higher density than in regions not bordering pores.^36^ Our lightsheet microscopy and histology (Figures 2C and 3A) also show high densities of chondrocytes haloing canals within the unmineralized cartilage; as a result, the location of canals could be identified from adjacent histological serial sections, even when the canal itself had not been captured in the section. Previous work on the little skate (*Leucoraja erinacea*, a batoid species) demonstrated that progenitor cells originating in the inner layer of the perichondrium were able to traverse cartilage canals and differentiate into chondrocytes deep within the unmineralized cartilage.^34^ Additionally, we observed strand-like structures (1–2 μm diameter) radiating from canals (Figures 3A, 3B, and 3F)—visible with multiple staining and imaging techniques—often connecting to the basal surfaces of tesserae and linking chondrocytes hundreds of microns away in a daisy-chain series. These odd structures—variously termed “*Saftbahnen*” or “interlacunar networks”—have also been observed in chimaera, chicken, mouse, rat, and human cartilage (see citations in ^25^^,^^70^) yet their function remains unknown and understudied. Their association with cartilage canals in elasmobranchs, however, indicates that *Saftbahnen* evolved early in vertebrates, perhaps in concert with canals, and therefore may be linked to chondrocyte nourishment, cartilage growth, and, later, mineralization.

Although matrix mineralization is clearly not a primary role for elasmobranch cartilage canals, our findings suggest they may still harbor the potential to promote calcification. In specimens of several different species, we observed endophytic masses within the cartilage (Figure 4D); these disordered mineralized features are hypothesized to result from damage or aberrant mineralization processes in elasmobranch skeletons.^26^ Our occasional observations of endophytic masses attached to canals (Figure 4D) suggest some link between the two features. Similarly, in several cases, we observed tesserae that had formed along the proximal ends of canals, tracking them for short distances along their dive into the matrix (Figure 4G). Myliobatiform stingrays are an extreme case of this phenomenon where long trabeculae, lined entirely by tesserae, form reinforcing struts in the jaws, mostly in species that eat hard-shelled prey (Figure 4H).^27^^,^^71^^,^^72^ We have found that the lumen of these trabeculae contains tissues similar to those in cartilage canals (Figure 4H), indicating that canals may have been structural precursors to trabeculae, therefore arguing they also played a role in the evolution of specialized diets in elasmobranchs.

Our discovery of persistent vascular cartilage canals as widespread features in the cartilage of adult rays and sharks raises a host of further questions regarding their ubiquity in elasmobranchs and other taxa, their development and evolution, and their physiological activity. Understanding the metabolic and mechanical factors that regulate the growth, invasion in cartilage, and persistence of these canals (i.e., the lack of endochondral ossification) should give clues to how some elasmobranchs attain massive skeletal masses^67^^,^^68^^,^^69^ or may have exceptionally long lifespans.^73^ In modern orthopedic medicine, the repair of joint cartilage poses a major challenge, primarily because it lacks a blood supply and therefore has only limited self-healing capacity.^74^^,^^75^ When devitalized mammalian articular cartilage plugs are grafted to vital cartilage plugs or implanted subcutaneously in nude mice, soft tissue canals can develop from the vital tissue, penetrating devitalized tissue.^74^^,^^75^^,^^76^^,^^77^ As cartilaginous fish skeletons continue growing throughout life, the study of their cartilage canals may provide keys to further biomedical methods for producing responsive implants for damaged cartilage, as well as immortalized (infinitely proliferative) cell lines and cutting-edge treatments for joint diseases.

### Limitations of the study

Our most detailed characterizations focus on adult specimens of the round stingray (*Urobatis halleri*). In contrast, other elasmobranch fish species were only included opportunistically (largely due to logistical challenges of sampling). Likewise, individual age and sex and the skeletal element studied could not be controlled during sampling. Therefore, as stated above, our allocation of certain elasmobranch species as lacking canals (Figure 4) is not a definitive demonstration of canal absence in those species nor their families. Our sampling method was adequate, however, to observe cartilage canals in every batoid species specimen we examined; therefore, those shark, agnathan, and chimera species we marked as lacking canals should be interpreted at least as having an extremely low and/or inconsistent canal density relative to all 17 batoid species studied. Additionally, the published data for non-chondrichthyan species is scant outside of those few tetrapod species from which most data on cartilage canals have been derived. These overall sampling restrictions created a patchy window into canal presence/absence across the elasmobranch and vertebrate phylogeny, limiting the conclusions we could draw regarding the sharing of developmental processes and molecular pathways.

## Resource availability

### Lead contact

Further information and requests for resources and reagents should be directed to and will be fulfilled by the Lead Contact, Mason Dean (mndean@cityu.edu.hk).

### Materials availability

This study did not generate new unique reagents.

### Data and code availability

• A representative portion of the Round stingray propterygium phosphotungstic acid-stained μCT and its associated binary segmentation has been deposited at OSF: https://doi.org/10.17605/OSF.IO/TXJ5Z and is publicly available as of the date of publication. Any additional information required to reanalyze the data reported in this paper is available from the lead contact upon request.
• This paper does not report original code.
• This paper does not report any other items.

## Acknowledgments

The authors thank Ocean Park Aquarium for donating specimens, Cheryl Wilga for generously sharing hard-won images of shark cartilage. The authors thank Daniel Fernando, Akshay Tanna, and the team at Blue Resources Trust (BRT, Sri Lanka), who facilitated access to mobulid specimens from local fisheries and their export to QUT and the Max Planck Queensland Centre (MPQC) for funding the mobulid specimen collection and shipment. Julien Lesseur, Daniel Werner, and Júlia Chaumel helped arrange the fantastic microCT data for Figure 2. Dominique Adriaens and Annabella Knab generously helped with histology. We thank the core facilities at Cornell University, specifically the College of Veterinary Medicine's Animal Health Diagnostic Center (AHDC) for embedding and staining the *R. bonasus* sample and the Aperio ScanScope Unit for imaging it. Additionally, we thank CityUHK Veterinary Diagnostic Laboratory for embedding and staining the *A. ocellatus* sample. Peter Fratzl and Adam Summers, as always, provided support and sharpened curiosity that drove the project forward, as did the lovely community of comparative skeletal biology researchers at the IAFSB/CCB/CCBB conferences over the past decade, since this project began. We thank generous funding of multiple sources: an HFSP Young Investigator’s Grant (RGY0067); the General Research Fund of the Research Grants Council of the Hong Kong SAR (GRF: 9043811); Joint Research Scheme with the Consulate General of France in Hong Kong (F-CityU103/21); a CORBEL grant (PID-2358), via the European Union’s Horizon 2020 (agreement 654248); CC Wu Cultural & Education Foundation Ltd Research Fund (9229187); Research Development Fund and Graduate Student Fund of the Faculty of Medical and Health Sciences, Waipapa Taumata Rau University of Auckland; and the U.S. National Science Foundation (grant no DGE – 2139899).

## Author contributions

B.F.: conceptualization, methodology, validation, formal analysis, investigation, resources, data curation, writing – original draft, writing – review and editing, visualization, project administration, and funding acquisition. R.S.: conceptualization, methodology, software, validation, formal analysis, investigation, data curation, writing – review and editing, and visualization. T.I.H.: methodology, validation, formal analysis, investigation, resources, data curation, writing – review and editing, and visualization. M.Y.-B.: methodology, validation, formal analysis, investigation, resources, data curation, writing – review and editing, and visualization. J.C.-H.: validation, formal analysis, investigation, writing – review and editing, and visualization. S.O.M.: validation, investigation, and writing – review and editing. E.J.G.: methodology, validation, formal analysis, investigation, resources, writing – review and editing, and visualization. K.L.: investigation, resources, and writing – review and editing. V.C.-A.: methodology, validation, resources, writing – review and editing, and project administration. S.R.M.: methodology, validation, resources, writing – review and editing, supervision, project administration, and funding acquisition. L.E.: methodology, validation, resources, writing – review and editing, and visualization. L.B.: methodology, validation, resources, writing – review and editing, supervision, project administration, and funding acquisition. M.D.-T.: conceptualization, methodology, validation, formal analysis, investigation, resources, data curation, writing – original draft, writing – review and editing, visualization, and supervision. D.B.: conceptualization, methodology, software, validation, formal analysis, investigation, resources, data curation, writing – review and editing, visualization, and supervision. M.J.B.: conceptualization, methodology, validation, formal analysis, investigation, resources, data curation, writing – original draft, writing – review and editing, visualization, and project administration. M.N.D.: conceptualization, methodology, validation, formal analysis, investigation, resources, data curation, writing – original draft, writing – review and editing, visualization, supervision, project administration, and funding acquisition.

## Declaration of interests

The authors declare no competing interests.

## STAR★Methods

### Key resources table

**Table:** 

REAGENT or RESOURCE	SOURCE	IDENTIFIER
**Chemicals, peptides, and recombinant proteins**		

Phosphotungstic acid (PTA)	Sigma Aldrich	CAS 12501–23–4, SKU: P4006-10G
DAPI	Invitrogen	SKU: D1306
Fast Green	Sigma Aldrich	CAS: 2353–45–9, SKU: F7252-25G
Dichloromethane	Sigma Aldrich	CAS: 75–09–2, SKU: 32222-2.5L
Dibenzylether	Merck	CAS: 103–50–4

**Deposited data**		

Cartilage canals in sharks and rays show that blood vessels can exist in mature cartilage without endochondral bone formation	This study	https://doi.org/10.17605/OSF.IO/TXJ5Z

**Experimental models: Organisms/strains**		

3 adult *Urobatis halleri* (round stingray)	Lyons et al.^78^	URHA36 (21.1 cm DW, M); URHA3 (11.5 cm DW, M); URHA27 (11.0 cm DW, M) *(MN Dean lab specimen IDs)*
1 adult *Rhinoptera bonasus* (cownose ray)	Bycatch: Commercial fishery, USA	RJBO06 (43 cm DW,F) *(MN Dean lab specimen ID)*
1 juvenile *Aetobatus ocellatus* (eagle ray)	Post-necropsy donation: Ocean Park Aquarium Veterinary Center	AEOC03 (55.8 cm DW, F) *(MN Dean lab specimen ID)*
1 juvenile *Carcharhinus brachyurus* (copper shark)	Bycatch: Commercial fishery, NZ	*C. brachyurus* 01 (144 cm TL, F) *(S McGlashan lab specimen ID)*
4 juvenile *Prionace glauca* (blue shark)	Bycatch: Commercial fishery, NZ	*P. glauca* 01 (130 cm TL, M); *P. glauca* 02 (141 cm TL, M); *P. glauca* 03 (129 cm TL, M); *P. glauca* 04 (120 cm TL, M) *(S McGlashan lab specimen ID)*
2 juvenile *Galeorhinus galeus* (school shark)	Bycatch: Commercial fishery, NZ	*G. galeus* 01 (73 cm TL, F); *G. galeus* 02 (77 cm TL, F) *(S McGlashan lab specimen ID)*
1 adult *Squalus chloroculus* (greeneye spurdog)	Bycatch: Commercial fishery, NZ	*S. chloroculus* 01 (75 cm TL, F) *(S McGlashan lab specimen ID)*

**Software and algorithms**		

Amira ZIB Edition	Zuse Institute Berlin Stalling et al.^79^	v. 2024.2
Python scikit-image	van der Walt et al.^80^	v. 0.26.0
Python SciPy	Virtanen et al.^81^	v. 1.18.1
FIJI	Schindelin et al.^82^	v. 2.9.0

**Other**		

Data from all other species not listed above are derived from re-analysis of images taken for other published studies (see Table S3)	N/A	N/A

### Experimental model and study participant details

The majority of data for this work is reanalyzed from images taken for previous studies (see Table S3 for sources); the following are the additional specimens examined specifically for this study.

#### *Urobatis halleri* specimens

Three Round stingray (*U. halleri*) specimens were used in this work, all donated from a previous study where they were collected in Seal Beach, California, USA.^78^ The tissues described below were excised from the donated specimens and kept for the current study, while other portions of the skeleton were used for other research (e.g.,^22^^,^^23^^,^^25^^,^^26^^,^^31^^,^^32^).

First, the left propterygium from a 21.1 cm disc width (DW) male was excised and used for phosphotungstic acid (PTA)-stained μCT scanning. Second, tissue from an 11.5 cm DW male was used to quantify pore morphometrics (Figure S2). A flat region of tesserae (∼6.2 × 2.0 mm) was excised from the side of the propterygium and most of the unmineralized cartilage removed, leaving predominantly the tessellated layer. Lastly, a cross section of propterygium from an 11.0 cm DW male was used to examine how canal visibility changes with tissue dehydration (Figure S1).

#### Galean and squalean shark specimens

Copper shark (*Carcharhinus brachyurus*), School shark (*Galeorhinus galeus*), Blue shark (*Prionace glauca*) and Greeneye spurdog (*Squalus chloroculus*) specimens were obtained as commercial bycatch off Mangawhai (*C. brachyurus*, *G. galeus)*, East Cape (*P. glauca*) and Whangaroa (*S. chloroculus*), New Zealand.

#### Eagle ray (*Aetobatus ocellatus*) and cownose ray (*Rhinoptera bonasus*) specimens

The Eagle ray (*A. ocellatus*) specimen was donated to the project from Ocean Park aquarium Hong Kong following its necropsy after dying of natural causes. The Cownose ray (*R. bonasus)* specimen was bycatch from commercial fishermen off of North Carolina, USA.

### Method details

#### Transmitted light microscopy

To measure pore morphometrics, a transmitted light microscopy image of the isolated tessellated layer of a Round stingray specimen (11.5 cm DW) was reduced to 8-bit, thresholded (Otsu, 0–250), inverted, eroded and dilated using Fiji.^82^ Python code, using *skimage.morphology.remove_small_objects*, removed noise. *Skimage.measure.label* and *.measure.regionprops* were used to label and extract the maximum Feret’s diameters of 28 pores.^80^ Centroid coordinates and *scipy.spatial.KDTree* were used to locate the nearest neighbor, and *scipy.spatial.distance.cdist* to calculate minimum Euclidean distance between pore edges.^81^

To examine how canal visibility changes with dehydration, the propterygium cross section from the 11.0 cm DW animal was placed in a drop of water in a Petri dish and observed under a Keyence Digital Microscope. Images of the entire cross section were taken sequentially as the sample dehydrated and a canal in the uncalcified matrix became apparent.

#### Phosphotungstic acid-stained microcomputed tomography (μCT)

The left propterygium of a Round stingray *U. halleri* specimen (21.1 cm DW) was excised and fixed in 75% ethanol. The propterygium was soaked in a 1% PTA solution for 36 h. Post-staining, the whole skeletal element was scanned in an RX Solutions EasyTom160 microCT scanner (Chavanod, France) at 80 kV source voltage and 125 μa source current with a final voxel size of 11 μm.

Reconstructed image slices were imported into Amira ZIB software^79^ where the data were downsampled to 22 μm voxel size and the cartilage canals were segmented (digitally dissected) semi-automatically using the magic wand tool, producing a label field of the canal network. An *Autoskeleton* module was attached to the resulting label field, from which all morphometric data were extracted. A Euclidean distance map was applied to the thinned label field from the *Autoskeleton* module, from which the distances from each canal to all others were determined, with the shortest pairwise distance for each canal taken as the nearest neighbor distance.

#### Unstained microcomputed tomography (μCT)

μCT data of air-dried, unstained Round stingray cartilage was re-rendered from previous studies.^23^^,^^36^

#### Lightsheet microscopy

Lightsheet microscopy was performed with a custom lightsheet microscope^83^ on cubes (5–6 mm) of PFA-fixed *Dasyatis pastinaca* propterygium, optically-cleared using ethyl cinnamate (ECi),^84^ as part of a study on tissue autofluorescence.^85^ The methods are as described there; however, we also explored combinations of different excitation laser lines and detection emission filters to create multi-spectral images (e.g., Figure 2C). This allowed us to distinguish canals/blood vessels autofluorescence (Ex: 561 nm; Em: 593LP, in red) from other structures, such as NADH autofluorescence from chondrocytes (Ex: 488 nm; Em: 525/50, in cyan).

#### Scanning electron microscopy

Images from backscatter electron microscopy (BSE-SEM: Figure 1F) and cryo-scanning electron microscopy (cryoSEM: Figure 2G) of Round stingray cartilage were reanalyzed from previous studies.^22^^,^^23^^,^^32^

#### Histology

Histological slides from Round stingray cartilage were reanalyzed from previous works, with methods as described previously.^23^^,^^25^

Similarly, histological slides (hematoxylin & eosin, Masson’s Trichrome) were reanalyzed from Devil ray cartilage (*Mobula mobular*, *M. thurstoni*, *M. tarapacana* and *M. eregoodoo*) from a previous study.^86^ Additionally, we reanalyzed fluorescent microscopy slides taken from the same animals, prepared for another forthcoming work. Briefly, non-decalcified cryosections (50 μm) were taken from the *M. thurstoni* and *M. tarapacana* samples, fully dehydrated in methanol, and stained using 0.2 μg/mL Fast Green in methanol solution after DAPI staining (1:1000). Sections were then cleared in dichloromethane and dibenzylether and imaged on an FLUOVIEW FV4000 confocal microscope (Evident, Tokyo, Japan) with a TOTO3 detector between 580 and 630 nm and DAPI detector between 430 and 470 nm.

The other species’ tissues examined specifically for this study were prepared as follows:

Copper shark, School shark, Blue shark and Greeneye spurdog pectoral girdle (coracoid bar) specimens were fixed in 4% PFA and stored in 0.1M PBS. Samples were decalcified in 20% EDTA in PBS at room temperature for 30 days and embedded in paraffin. Serial cross-sections (8 μm) were mounted and stained with Masson’s Trichrome (MT). All shark specimens were processed and stained at the University of Auckland Histology Services. Entire cross-sections were imaged using an Olympus SLIDEVIEW VS200 slide-scanner (Olympus, Version 4.1).

For Eagle ray and Cownose ray specimens, the jaws were removed and fixed in 4% PFA for 24 h before being stored in phosphate-buffered saline. Samples were decalcified in 10% EDTA until fully decalcified and embedded in paraffin. Samples were cut into 5 μm cross-sections and auto-stained with Masson’s trichrome. The Eagle ray was processed and stained at the City University of Hong Kong’s Veterinary Diagnostic Laboratory while the Cownose ray was processed and stained at the Cornell Animal Health Diagnostic Center. The *A. ocellatus* slide was imaged on a Nikon Ni-E upright microscope. The *R. bonasus* slide was imaged on a Leica Aperio ScanScope CS.

### Quantification and statistical analysis

There are no quantification or statistical analyses to include in this study.
