# Supplementary material for: Shark and ray cartilage canals show blood vessels can exist in mature cartilage without endochondral bone formation
Source: iScience. 2026 Oct 31;29(11):117871. doi: 10.1016/j.isci.2026.117871 (PMC13743901; doi:10.1016/j.isci.2026.117871)
Supplement: Supplementary file 1 [file mmc1.pdf]

iScience, Volume *29*

Supplemental information

Shark and ray cartilage canals show blood vessels can exist in mature cartilage without endochondral bone formation

Benjamin Flaum, Ronald Seidel, Maximus Yeatman-Biggs, Theda I. Hinrichs, Jana Ciecierska-Holmes, Salman O. Matan, Emilio J. Gualda, Kady Lyons, Victoria Camilieri-Asch, Susan R. McGlashan, Laura Ekstrom, Lawrence Bonassar, Mélanie Debiais-Thibaud, Daniel Baum, Michael J. Blumer, and Mason N. Dean

Supplementary figures and tables.

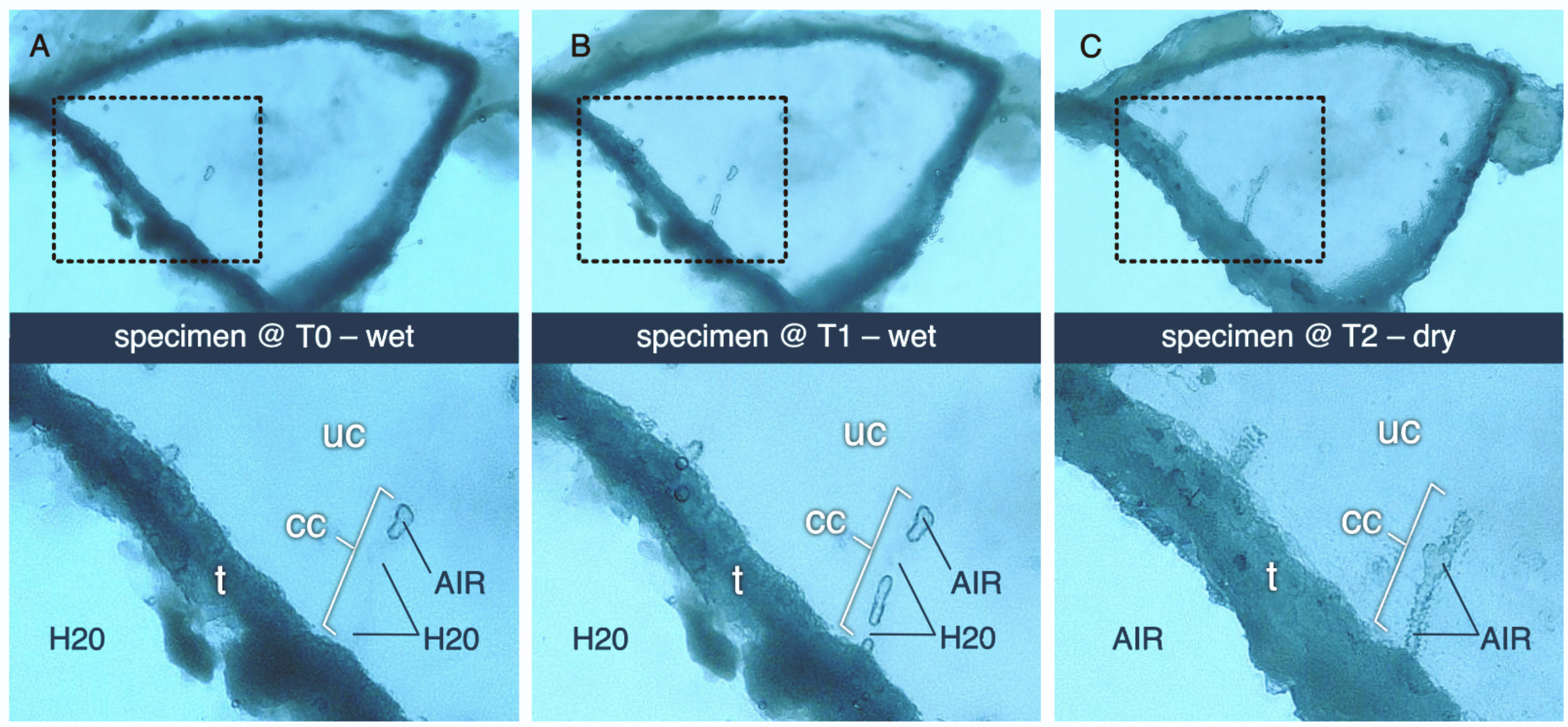

Figure S1: Light micrographs showing cartilage canal (cc) visibility changing with dehydration, in a cross section of propterygium from the Round stingray U. halleri (11 cm DW). The dashed boxes in the upper row indicate the regions of unmineralized cartilage (uc) and tesserae (t) magnified in the bottom row, shown from left to right over three consecutive time points (T0-2). A) Fresh cut (hydrated) specimen, where a small amount of air has been introduced into the distal end of the canal. B-C) As the specimen dries, air gradually fills the canal back to its proximal end at the tesseral layer, revealing the canal's full extent.

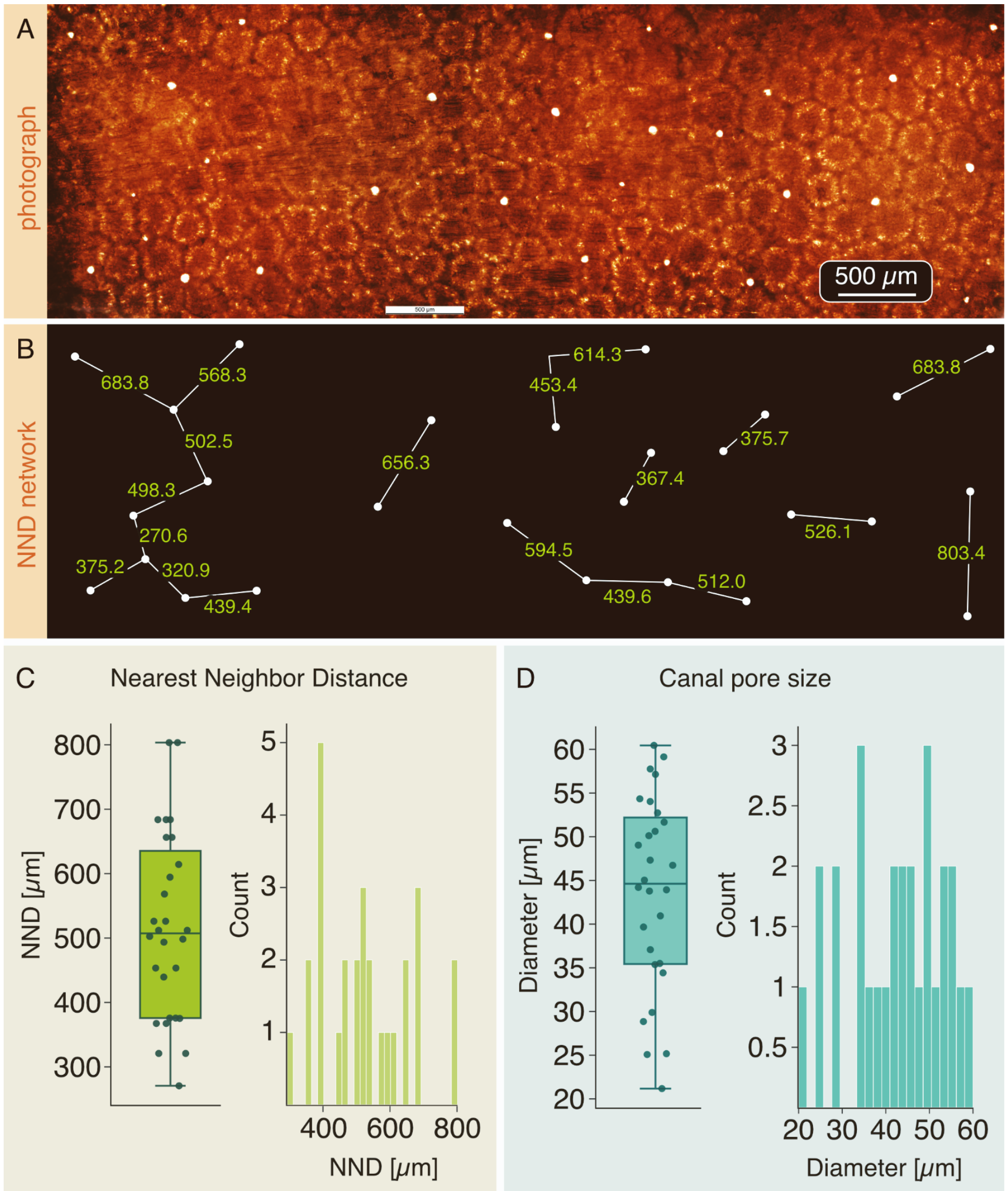

Figure S2: Canal pore morphometrics from Round stingray *U. halleri*; A) A backlit light micrograph of tesserae in a flat region of interest of the propterygium, with most of the unmineralized cartilage removed; pores are visible as white spots. B) The isolated network of canal pores showing the calculated nearest neighbor distances (NND, pore edge-to-edge). C-D) Nearest neighbor distances and canal pore size (maximum Feret Diameter) from 28 canal pores. See also Table S1. (Scalebars: A = 500μm)

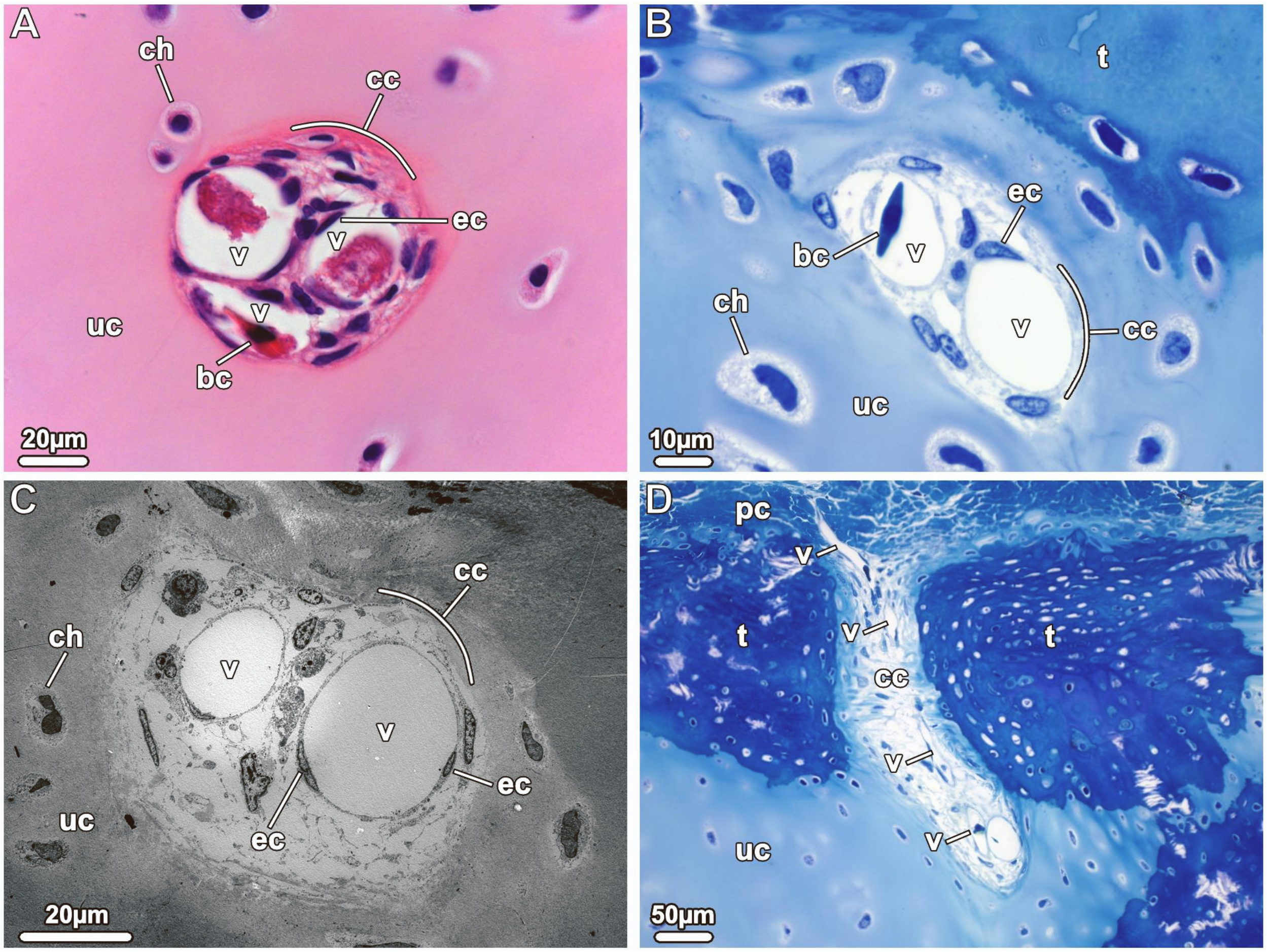

Figure S3: Light (LM) and transmission electron microscopy (TEM) of Round stingray *U. halleri* cartilage confirm that the cartilage canals of elasmobranch fishes contain blood vessels originating from the perichondrium. (A, B) LM. HE and TB staining. (C) TEM. Cross sections through a cartilage canal (cc) embedded in the unmineralized cartilage matrix (uc) of a skeletal element. The canals contain blood vessels (v) lined by a single layer of flat endothelial cells (ec). In (A, B), red blood cells (bc) can be seen in the lumen of vessels (v). (D) LM. TB staining of a longitudinal section of a cartilage canal (cc) originating from the perichondrium (pc) and extending deep into the unmineralized cartilage (uc). Note the course of the blood vessel (v), which emerges from the perichondrium and runs through the entire canal. ch = chondrocytes; t = tesserae. (Scalebars: A,C = 20µm; B = 10µm; D = 50µm)

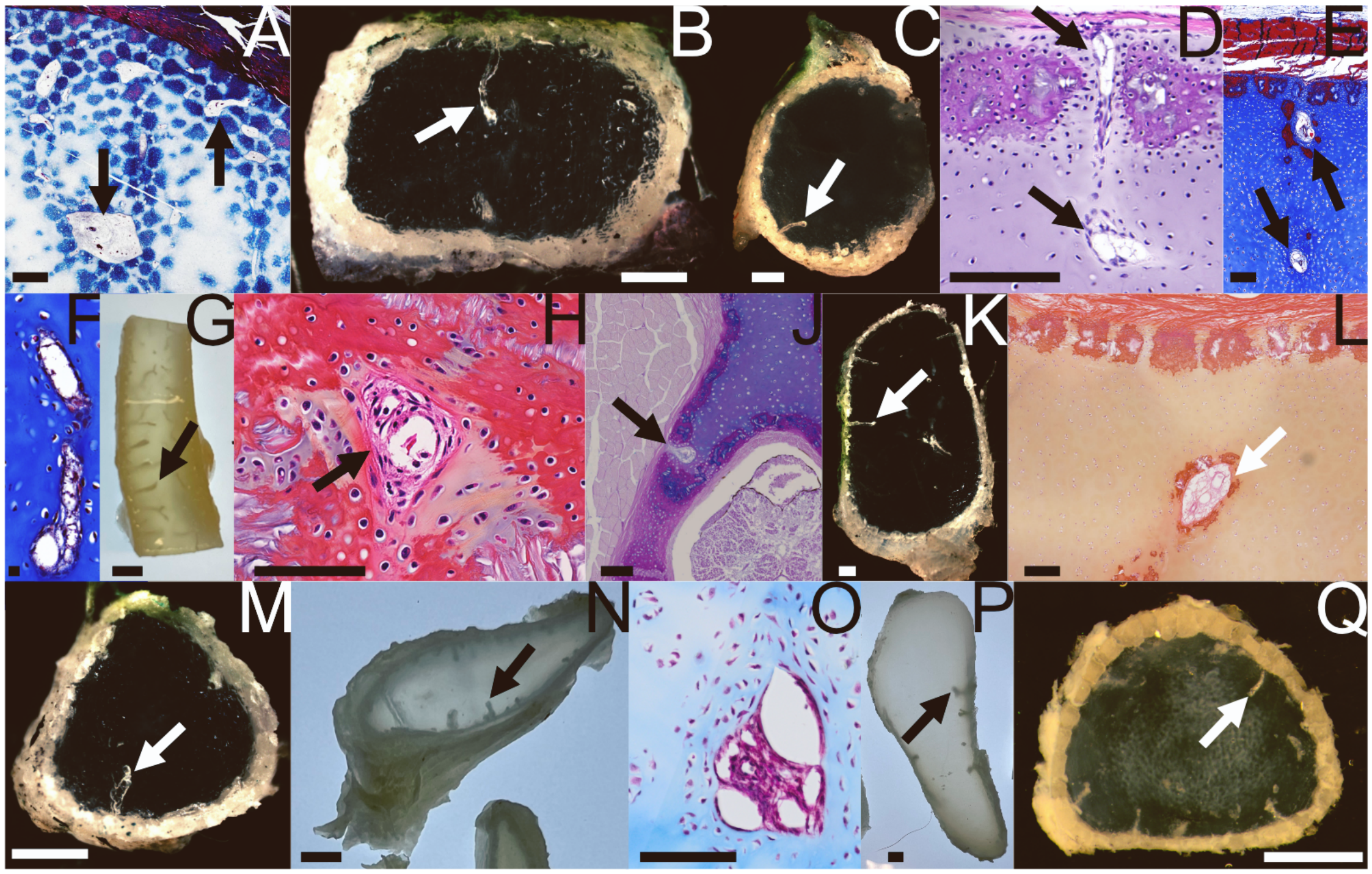

Figure S4: Elasmobranch canals from those examined species not featured in Figure 4. Arrows indicate cartilage canals. A) *Aetobatus ocellatus*, B) *Hypanus sabinus*, C) *Pteroplatytrygon violacea*, D) *Mobula mobular*, E) *Mobula thurstoni*, F) *Mobula eregoodoo*, G) *Raja binoculata*, H) *Raja asterias*, J) *Raja clavata*, K) *Raja eglanteria*, L) *Rhina ancylostoma*, M) *Narcine bancroftii*, N) *Mustelus canis*, O) *Prionace glauca*, P) *Alopias vulpinus*, Q) *Chiloscyllium plagiosum*. In all images, canals are ~30-50 µm in diameter; panels A,F,L show canals with partial or complete lining by tesserae. The figure includes images of macroscopic skeletal sections (B,C,G,K,M,N,P,Q) and close-up images of individual canals (A,D,E,F,H,J,L,O); canals are sectioned longitudinally in all cases, except H and O. See Table S3 for the particular skeletal elements examined. Scalebars: A,B,C,M = 500µm; F = 10µm; D,E,H,J,L,O = 100µm; K,N,P,Q = 2mm)

Table S1: Canal pore morphometrics, derived from measurements from transmitted light micrographs from a 6.2x2.0mm ROI of Round stingray *U. halleri* tessellated cartilage (see also Fig. S2).

	**Mean**	**SD**	**Min**	**Max**
Pore diameter (μm)	43.6	11.1	21.2	60.5
Nearest neighbor distance (μm)	515.7	144.3	270.6	803.4

Table S2: Canal morphometrics, derived from an Amira-based quantification of PTA-stained μCT data of a complete Round stingray *U. halleri* propterygium (see Fig. 2A,B).

	**Mean**	**SD**	**Min**	**Max**
Mean radius (μm)	12.05	2.66	10.99	30.24
Curved length (μm)	453.29	337.89	96.14	1766.42
Tortuosity	1.08	0.08	1	1.66
Distance between canals (μm)	571.42	176.79	115.13	1280.16

Total number of canals measured: $n = 408$; Ratio of canal to cartilage volume: 0.00296 (0.296%)

Table S3: Elasmobranch samples examined, listed by order and major group (shark or batoid; see Fig. 4). Canal presence is indicated in the fourth column: species are marked as having canals if at least one canal was unequivocally observed across numerous specimens. Subsequent columns list the anatomical location of observations, the study methods used, and literature sources, when data were reanalyzed from previous studies.

**Species**	**Order**	**Shark/batoid**	**Canals?**	**Location**	**Methods**	**Source**
Urobatis halleri	Myliobatiformes	Batoid	Y	PT[1–5] J[6]	H[1,2], cSEM[6], BSE-SEM[7], P[3], SR-µCT[4], µCT, LS[5]	[1–6] Current study
Aetobatus ocellatus	Myliobatiformes	Batoid	Y	J	H	BF
Dasyatis pastinaca	Myliobatiformes	Batoid	Y	PG	H	[5]
Hypanus sabinus	Myliobatiformes	Batoid	Y	PG[8],J[9,10]	P	[8–10]
Pteroplatytrygon violacea	Myliobatiformes	Batoid	Y	PG	P	[8,11]
Mobula mobular	Myliobatiformes	Batoid	Y	CH	H	[12]
Mobula thurstoni	Myliobatiformes	Batoid	Y	CH	H	[12]
Mobula tarapacana	Myliobatiformes	Batoid	Y	CH	H	[12]
Mobula eregoodoo	Myliobatiformes	Batoid	Y	CH	H	[12]
Rhinoptera bonasus	Myliobatiformes	Batoid	Y	J,PG	H	BF+MND
Leucoraja erinacea	Rajiformes	Batoid	Y	HY	P	[9,10,13]
Raja binoculata	Rajiformes	Batoid	Y	HY	P	[9,10]
Raja asterias	Rajiformes	Batoid	Y	J	H	[14]
Raja clavata	Rajiformes	Batoid	Y	CH?[15],S[13]	I	[13,15]
Raja eglanteria	Rajiformes	Batoid	Y	PG	P	[8]
Rhina ancylostoma	Rhinopristiformes	Batoid	Y	NA	H	[16]
Narcine bancroftii	Torpediniformes	Batoid	Y	HY	P	[8]
Carcharhinus brachyurus	Carcharhiniformes	Shark (Galea)	N	PG	H	MYB+SM
Carcharhinus plumbeus	Carcharhiniformes	Shark (Galea)	Y	HY	P	[9,10]
Galeorhinus galeus	Carcharhiniformes	Shark (Galea)	Y	PG	H	MYB+SM
Mustelus canis	Carcharhiniformes	Shark (Galea)	Y	HY	P	[9,10]
Prionace glauca	Carcharhiniformes	Shark (Galea)	Y	PG	H	MYB+SM
Scyliorhinus canicula	Carcharhiniformes	Shark (Galea)	N	J,HY,NA,PG	H	[13,14,17]
Scyliorhinus retifer	Carcharhiniformes	Shark (Galea)	N	J,HY	P	[1,18]
Alopias vulpinus	Lamniformes	Shark (Galea)	Y	HY	P	[9,10]
Cetorhinus maximus	Lamniformes	Shark (Galea)	N?	B	P	[19]
Isurus oxyrinchus	Lamniformes	Shark (Galea)	Y	HY	P	[9,10]
Chiloscyllium plagiosum	Orectolobiformes	Shark (Galea)	Y	HY	P	[9,10]
Hexanchus griseus	Hexanchiformes	Shark (Squalea)	N	HY	P	[9,10]
Notorynchus cepedianus	Hexanchiformes	Shark (Squalea)	N	HY	P	[9,10]
Somniosus pacificus	Squaliformes	Shark (Squalea)	N	HY	P	[9,10]
Squalus acanthias	Squaliformes	Shark (Squalea)	N	HY	P	[9,10]
Squalus chloroculus	Squaliformes	Shark (Squalea)	N	PG	H	MYB+SM

Squatina squatina	Squatiniformes	Shark (Squalea)	Y	HY	P	[9,10]

<u>*Abbreviation key*</u>*:*

Anatomical location: J=Jaws; CH=Chondrocranium; HY=Hyoid arch; NA=Neural arch; PG=Pectoral/pelvic girdle (including propterygium, fin radials); S=synarcual (fused vertebrae)

Methods: BSE-SEM=backscatter electron SEM; cSEM=cryo-SEM; H=histology; I=Illustration; LS=Light-sheet microscopy; P=Skeleton section photography; SR-μCT=Synchrotron μCT

Source: Literature listed by numbered citation, personal observations listed by author initials: BF=Benjamin Flaum; MND=Mason Dean; MYB=Maximus Yeatman-Biggs; SM= Susan McGlashan

Table S4: Non-elasmobranch groups, listed by clade and family (see Fig. 4), with canal presence indicated. Tetrapod vertebrates are far more studied than other groups, with a focus on several model species (e.g. chicken, dog, horse, human, mouse). In contrast, literature for non-amniote examples is sparse. A group's designation —especially in the non-tetrapod examples— should therefore not be taken as indication that all members of the group have or do not have canals. Note, in our literature survey, the many published investigations of actinopterygian skeletal histology typically do not mention cartilage canals; the study[20] of the marine teleost *Sparus auratus*, however, specifically notes the lack of canals in the cartilage.

Clade	Example families	Canals?	Sources
Agnatha	Myxinidae (hagfish)	N	[21–23]
Agnatha	Petromyzontidae (lamprey)	N	[24,25]
Holocephali	Chimaeridae (chimaera)	N	[13,18]
Actinopterygii	Acipenseridae (sturgeon)	Y	[15]
Actinopterygii	Sparidae (seabream)	N	[20]
Amphibia	Ranidae (frog)	Y	[26]
Reptilia	Varanidae (lizard)	Y	[27,28]
Reptilia	Dermochelyidae (turtle)	Y	[29]
Reptilia	Phasianidae (chicken)	Y	[30–33]
Mammalia	Hominidae (human)	Y	[34–37]
Mammalia	Muridae (mice)	Y	[38–41]
Mammalia	Equidae (horse)	Y	[42–44]
Mammalia	Canidae (dog)	Y	[45–47]
Mammalia	Leporidae (rabbit)	Y	[41,48–50]
Mammalia	Suidae (pig)	Y	[51–53]

Literature cited

1. Seidel, R., Lyons, K., Blumer, M., Zaslansky, P., Fratzl, P., Weaver, J.C., and Dean, M.N. (2016). Ultrastructural and developmental features of the tessellated endoskeleton of elasmobranchs (sharks and rays). J. Anat. *229*, 681–702.

2. Seidel, R., Blumer, M., Pechriggl, E.-J., Lyons, K., Hall, B.K., Fratzl, P., Weaver, J.C., and Dean, M.N. (2017). Calcified cartilage or bone? Collagens in the tessellated endoskeletons of cartilaginous fish (sharks and rays). J. Struct. Biol. *200*, 54–71.

3. Seidel, R., Blumer, M., Zaslansky, P., Knötel, D., Huber, D.R., Weaver, J.C., Fratzl, P., Omelon, S., Bertinetti, L., and Dean, M.N. (2017). Ultrastructural, material and crystallographic description of endophytic masses - A possible damage response in shark and ray tessellated calcified cartilage. J. Struct. Biol. *198*, 5–18.

4. Chaumel, J., Schotte, M., Bizzarro, J.J., Zaslansky, P., Fratzl, P., Baum, D., and Dean, M.N. (2020). Co-aligned chondrocytes: Zonal morphological variation and structured arrangement of cell lacunae in tessellated cartilage. Bone *134*, 115264.

5. Chaumel, J., Marsal, M., Gómez-Sánchez, A., Blumer, M., Gualda, E.J., de Juan, A., Loza-Alvarez, P., and Dean, M.N. (2021). Autofluorescence of stingray skeletal cartilage: hyperspectral imaging as a tool for histological characterization. Discover Materials *1*, 16.

6. Dean, M.N., Mull, C.G., Gorb, S.N., and Summers, A.P. (2009). Ontogeny of the tessellated skeleton: insight from the skeletal growth of the round stingray *Urobatis halleri*. J. Anat. *215*, 227–239.

7. Seidel, R., Roschger, A., Li, L., Zhang, Q., Yin, J., Yang, T., Weaver, J.C., Fratzl, P., Roschger, P., and Dean, M.N. (2019). Mechanical properties of stingray tesserae: High-resolution correlative analysis of mineral density and indentation moduli in tessellated cartilage. Acta Biomater. *96*, 421–435.

8. Macesic, L.J., and Summers, A.P. (2012). Flexural stiffness and composition of the batoid propterygium as predictors of punting ability. J. Exp. Biol. *215*, 2003–2012.

9. Wilga, C., Dumont, E., and Ferry, L. (2024). The effect of tessellation on stiffness in the hyoid arch of elasmobranchs. J. Morphol. *285*, e21681.

10. Wilga, C., Ferry, L., and Dumont, E. (2024). The effect of jaw suspension on cartilage strength in elasmobranchs. J. Morphol. *285*, e21745.

11. Dean, M.N., Flaum, B., and Debiais-Thibaud, M. (2024). Cartilaginous fish skeletal tissues. In Encyclopedia of Fish Physiology (Elsevier), pp. 452–459.

12. Hinrichs, T., Zhong, J., Chang, C.-W., Ramajeyam, G., Anusha, N., Amini, S., Dean, M.N., Collin, S.P., Hutmacher, D.W., Klein, T., et al. (2026). Biomechanics and composition of fixed neurocranial tessellated cartilage from the neurocranium of four mobulid ray species with different depth ranges. J. R. Soc. Interface *10.1098/rsif.[1331]*.

13. Debiais-Thibaud, M. (2018). The evolution of endoskeletal mineralisation in chondrichthyan fish. In Evolution and Development of Fishes, Z. Johanson, C. Underwood, and M. Richter, eds. (Cambridge University Press), pp. 110–125.

14. Enault, S., Auclair, C., Adnet, S., and Debiais-Thibaud, M. (2016). A complete protocol for the preparation of chondrichthyan skeletal specimens. J. Appl. Ichthyol. *32*, 409–415.

15. Leydig, F. (1857). Lehrbuch der Histologie des Menschen und der Thiere.

16. Berio, F., Broyon, M., Enault, S., Pirot, N., López-Romero, F.A., and Debiais-Thibaud, M. (2021). Diversity and evolution of mineralised skeletal tissues in chondrichthyans. Front. Ecol. Environ. *9*, 660767.

17. Berio, F., Broyon, M., Enault, S., Pirot, N., López-Romero, F.A., and Debiais-Thibaud, M. (2021). Diversity and evolution of mineralized skeletal tissues in chondrichthyans. Front. Ecol. Evol. *9*. 10.3389/fevo.2021.660767.

18. Seidel, R., Blumer, M., Chaumel, J., Amini, S., and Dean, M.N. (2020). Endoskeletal mineralization in chimaera and a comparative guide to tessellated cartilage in chondrichthyan fishes (sharks, rays and chimaera). J. R. Soc. Interface *17*, 20200474.

19. Li, T., Schindler, M., Paskin, M., Surapaneni, V.A., Scott, E., Hauert, S., Payne, N., Cade, D.E., Goldbogen, J.A., Mollen, F.H., et al. (2025). Functional models from limited data: A parametric and multimodal approach to anatomy and 3D kinematics of feeding in basking sharks (*Cetorhinus maximus*). Anat. Rec. (Hoboken). 10.1002/ar.25693.

20. Estêvão, M.D., Silva, N., Redruello, B., Costa, R., Gregório, S., Canário, A.V.M., and Power, D.M. (2011). Cellular morphology and markers of cartilage and bone in the marine teleost *Sparus auratus*. Cell Tissue Res. *343*, 619–635.

21. Ota, K.G., Fujimoto, S., Oisi, Y., and Kuratani, S. (2013). Late development of hagfish vertebral elements. J. Exp. Zool. B Mol. Dev. Evol. *320*, 129–139.

22. Ota, K.G., Fujimoto, S., Oisi, Y., and Kuratani, S. (2011). Identification of vertebra-like elements and their possible differentiation from sclerotomes in the hagfish. Nat. Commun. *2*, 373.

23. Robson, P., Wright, G.M., and Keeley, F.W. (2000). Distinct non-collagen based cartilages comprising the endoskeleton of the Atlantic hagfish, *Myxine glutinosa*. Anat. Embryol. (Berl.) *202*, 281–290.

24. Root, Z.D., Jandzik, D., Gould, C., Allen, C., Brewer, M., and Medeiros, D.M. (2023). Cartilage diversification and modularity drove the evolution of the ancestral vertebrate head skeleton. Evodevo *14*, 8.

25. Morel, C. (2014). Etude de la squelettogenèse chez la lamproie marine (*Petromyzon marinus*).

26. Rozenblut, B., and Ogielska, M. (2005). Development and growth of long bones in European water frogs (Amphibia: Anura: Ranidae), with remarks on age determination. J. Morphol. *265*, 304–317.

27. Haines, R. (1942). The evolution of epiphyses and of endochondral bone. Biol. Rev. Camb. Philos. Soc.

28. de Buffrénil, V., Ineich, I., and Böhme, W. (2005). Comparative data on epiphyseal development in the family varanidae. J. Herpetol. *39*, 328–335.

29. Rhodin, A.G.J., Ogden, J.A., and Conlogue, G.J. (1981). Chondro-osseous morphology of *Dermochelys coriacea*, a marine reptile with mammalian skeletal features. Nature *290*, 244–246.

30. Blumer, M.J.F., Longato, S., and Fritsch, H. (2004). Cartilage canals in the chicken embryo are involved in the process of endochondral bone formation within the epiphyseal growth plate. Anat. Rec. *279A*, 692–700.

31. Eslaminejad, M.R.B., Valojerdi, M.R., and Yazdi, P.E. (2006). Computerized three-dimensional reconstruction of cartilage canals in chick tibial chondroepiphysis. Anatomia, Histologia, Embryologia: Journal of Veterinary Medicine Series C *35*, 247–252.

32. Lutfi, A.M. (1970). Mode of growth, fate and functions of cartilage canals. J. Anat. *106*, 135–145.

33. Blumer, M.J.F., Fritsch, H., Pfaller, K., and Brenner, E. (2004). Cartilage canals in the chicken embryo: ultrastructure and function. Anat. Embryol. *207*, 453–462.

34. Chappard, D., Alexandre, C., and Riffat, G. (1986). Uncalcified cartilage resorption in human fetal cartilage canals. Tissue Cell *18*, 701–707.

35. Haines, R.W. (1933). Cartilage canals. J. Anat. *68*, 45.

36. Levene, C. (1964). The patterns of cartilage canals. J. Anat. *98*, 515–538.

37. Rodríguez, J.I., Delgado, E., and Paniagua, R. (1985). Multivacuolated cells in human cartilage canals. Acta Anat. (Basel) *124*, 54–57.

38. Blumer, M.J.F., Longato, S., Schwarzer, C., and Fritsch, H. (2007). Bone development in the femoral epiphysis of mice: the role of cartilage canals and the fate of resting chondrocytes. Dev. Dyn. *236*, 2077–2088.

39. Blumer, M.J.F., Longato, S., and Fritsch, H. (2008). Localization of tartrate-resistant acid phosphatase (TRAP), membrane type-1 matrix metalloproteinases (MT1-MMP) and macrophages during early endochondral bone formation. J. Anat. *213*, 431–441.

40. Cole, A.A., and Wezeman, F.H. (1985). Perivascular cells in cartilage canals of the developing mouse epiphysis. Am. J. Anat. *174*, 119–129.

41. Kugler, J.H., Tomlinson, A., Wagstaff, A., and Ward, S.M. (1979). Role of cartilage canals in the formation of secondary centers of ossification. J. Anat. *129*, 493–506.

42. Hellings, I.R., Dolvik, N.I., Ekman, S., and Olstad, K. (2017). Cartilage canals in the distal intermediate ridge of the tibia of fetuses and foals are surrounded by different types of collagen. J. Anat. *231*, 615–625.

43. Olstad, K., Hendrickson, E.H.S., Carlson, C.S., Ekman, S., and Dolvik, N.I. (2013). Transection of vessels in epiphyseal cartilage canals leads to osteochondrosis and osteochondrosis dissecans in the femoro-patellar joint of foals; a potential model of juvenile osteochondritis dissecans. Osteoarthritis Cartilage *21*, 730–738.

44. Olstad, K., Ytrehus, B., Ekman, S., Carlson, C.S., and Dolvik, N.I. (2007). Early lesions of osteochondrosis in the distal tibia of foals. J. Orthop. Res. *25*, 1094–1105.

45. Di Giancamillo, A., Andreis, M.E., Taini, P., Veronesi, M.C., Di Giancamillo, M., and Modina, S.C. (2016). Cartilage canals in newborn dogs: histochemical and immunohistochemical findings. Eur. J. Histochem. *60*, 2701.

46. Wilsman, N.J., and Van Sickle, D.C. (1970). The relationship of cartilage canals to the initial osteogenesis of secondary centers of ossification. Anat. Rec. *168*, 381–391.

47. Wilsman, N.J., and Van Sickle, D.C. (1972). Cartilage canals, their morphology and distribution. Anat. Rec. *173*, 79–93.

48. Roach, H.I. (1999). Association of matrix acid and alkaline phosphatases with mineralization of cartilage and endochondral bone. Histochem. J. *31*, 53–61.

49. Doschak, M.R., Cooper, D.M.L., Huculak, C.N., Matyas, J.R., Hart, D.A., Hallgrimsson, B., Zernicke, R.F., and Bray, R.C. (2003). Angiogenesis in the distal femoral chondroepiphysis of the rabbit during development of the secondary centre of ossification. J. Anat. *203*, 223–233.

50. Melton, J.T.K., Clarke, N.M.P., and Roach, H.I. (2006). Matrix metalloproteinase-9 induces the formation of cartilage canals in the chondroepiphysis of the neonatal rabbit. J. Bone Joint Surg. Am. *88 Suppl 3*, 155–161.

51. Finnøy, A., Olstad, K., and Lilledahl, M.B. (2017). Non-linear optical microscopy of cartilage canals in the distal femur of young pigs may reveal the cause of articular osteochondrosis. BMC Vet. Res. *13*, 270.

52. Ytrehus, B., Andreas Haga, H., Mellum, C.N., Mathisen, L., Carlson, C.S., Ekman, S., Teige, J., and Reinholt, F.P. (2004). Experimental ischemia of porcine growth cartilage produces lesions of osteochondrosis. J. Orthop. Res. *22*, 1201–1209.

53. Ytrehus, B., Ekman, S., Carlson, C.S., Teige, J., and Reinholt, F.P. (2004). Focal changes in blood supply during normal epiphyseal growth are central in the pathogenesis of osteochondrosis in pigs. Bone *35*, 1294–1306.
